# In Reply: Safety and Effectiveness of an Enhanced Recovery Protocol in Patients Undergoing Burr Hole Evacuation for Chronic Subdural Hematoma

**DOI:** 10.1227/neu.0000000000003235

**Published:** 2024-10-24

**Authors:** Victor E. Staartjes, Carlo Serra, Luca Regli

**Affiliations:** Machine Intelligence in Clinical Neuroscience & Microsurgical Neuroanatomy (MICN) Laboratory, Department of Neurosurgery, Clinical Neuroscience Center, University Hospital Zurich, University of Zurich, Zurich, Switzerland

**Keywords:** Chronic subdural hematoma, Hygroma, Burr hole, ERAS, Recovery

To the Editor:

We read with interest the letter to the editor^1^ by our colleague Dr Ribeiro da Costa on our recent article^2^ reporting our initial experience with an enhanced recovery after surgery (ERAS) protocol vs a historical control group of patients undergoing burr-hole evacuation of chronic subdural hematoma. To recapitulate, the main protocol differences distinguishing our ERAS protocol from our standard protocol include:

1. Thromboprophylaxis is started 6 hours postoperatively, instead of after 48 hours of drain removal
2. Patients postoperatively rest with the upper body 30° elevated, instead of in flat supine position
3. After 6 hours, patients are allowed to sit up and mobilize, instead of strict bed rest until drain removal
4. When sitting up or mobilizing, the drain does not need to be clamped, instead of it having to be clamped shut before
5. The drainage bag is generally held at heart level, instead of fixated to the bed at neck or shoulder level
6. Oral anticoagulants and antiaggregants are started again one day after suture/staple removal (which is performed on postoperative day 8), instead of only after the clinical follow-up visit 4 to 8 weeks after the surgery

We are grateful to our colleagues for acknowledging the importance of continuing evidence-based evaluation and improvement of the treatments we provide to patients with common neurosurgical diseases such as chronic subdural hematoma. Dr Ribeiro da Costa and colleagues appear to be in full agreement with the findings of our study. In addition, they mention the recently published, single-center randomized GET-UP trial^3,4^ including its 1-year results, demonstrating that in their cohort, early (<48 hours elevation of bed head) vs late (>48 hours recumbent position) mobilization led to a similar 1-month and 1-year recurrence rate with a markedly improved rate of Glasgow Coma Scale Extended ≥5 in the early mobilization group. In addition, the trial demonstrated a reduction in medical complications (infection, seizure, thrombotic event), which our study was not powered to assess. We commend our colleagues for conducting the GET-UP trial, contributing higher-level evidence of the effectiveness and safety of early mobilization and helping to dispel the mythos of early verticalization in this patient population being dangerous. Our 2 studies complement each other and will hopefully lead to further studies and a change of practice and guidelines.

ERAS is about implementing a holistic, multidisciplinary approach to improving recovery. Mobilization certainly is one major aspect (not only early postoperative verticalization but also implementation of early physiotherapy and in some cases of prehabilitation, too) of ERAS, but other measures such as nutritional assessment and optimization, thromboprophylaxis, opiate-free (or general anesthesia free) surgical procedures, and optimizing drain management represent classic ERAS elements.^5^ Our studies provide a stepping stone for higher-level trials evaluating the safety and effectiveness of such more complex ERAS protocols.

## Ethical Review

The scientific workup is approved by the local ethics review board (Kantonale Ethikkommission Zürich PB-2017-00093) and registered internationally at clinicaltrials.gov (NCT01628406). This report is compiled according to the Strengthening the Reporting of Observational Studies in Epidemiology statement.
